# Lower serum LDL-C levels are associated with poor prognosis in severe fever with thrombocytopenia syndrome: a single-center retrospective cohort study

**DOI:** 10.3389/fmicb.2024.1412263

**Published:** 2024-06-24

**Authors:** Shuai Guo, Qing Dong, Maomei Zhang, Lirui Tu, Yunjun Yan, Shougang Guo

**Affiliations:** ^1^Department of Neurology, Shandong Provincial Hospital, Shandong University, Jinan, China; ^2^Department of Neurology, Shandong Provincial Hospital Affiliated to Shandong First Medical University, Jinan, China; ^3^Department of Infectious Diseases, Shandong Public Health Clinical Center, Jinan, China; ^4^Shandong University of Traditional Chinese Medicine, Jinan, China; ^5^Jinan Dian Medical Laboratory Co., Ltd., Jinan, China

**Keywords:** SFTS, lipid profiles, LDL-C, prognosis, risk factors

## Abstract

**Background:**

Severe fever with thrombocytopenia syndrome (SFTS) is an emerging infectious disease triggered by a novel bunyavirus (SFTSV). Characterized by fever, thrombocytopenia, leukocytopenia, and multiple organ dysfunction manifestations, its primary mode of transmission is through tick bites. Despite the critical role of lipid metabolism in viral infections, the role of lipids in SFTS remains unclear.

**Methods:**

This retrospective study analyzed 602 patients with SFTS treated at the Shandong Public Health Clinical Center from January 2021 to December 2023. Based on the endpoint events, patients were classified into survival (S) and death (D) groups. The S group was further classified into non-critical (non-C) and critical (C) groups based on symptoms. All patients were followed up for at least 28 days after admission. Propensity score matching, multivariable logistic regression, survival analysis, time trend analysis, and mediation analysis were conducted to assess the association between LDL-C levels and prognosis in SFTS.

**Results:**

The serum LDL-C levels on admission were significantly lower in the D and C groups than in the S and non-C groups. The logistic regression models indicated a potential association between LDL-C levels and a poor prognosis in SFTS. The restricted cubic spline showed a unidirectional trend between LDL-C levels and mortality, with a cutoff value of 1.59 mmol/L. The survival analysis revealed higher and earlier mortality in the low-LDL-C group than in the high-LDL-C group. The trends over 28 days post-admission showed that the serum LDL-C levels gradually increased in SFTS, with a favorable prognosis. Finally, the mediation analysis indicated that low LDL-C levels are associated with mortality through poor hepatic, cardiac, and coagulation functions.

**Conclusion:**

Low LDL-C levels are potentially associated with a poor prognosis in SFTS.

## 1 Introduction

Severe fever with thrombocytopenia syndrome (SFTS), a rapidly progressive infectious disease caused by the SFTS virus (SFTSV) (Liu et al., 2014; Li et al., 2018), is mainly reported in East Asia, particularly in China, South Korea, and Japan (Yu et al., 2011; Kim et al., 2018; Kobayashi et al., 2020). It is associated with a high mortality rate (up to 20%) if not promptly diagnosed and treated (Yu et al., 2011; Liu et al., 2014; Li et al., 2018; Kobayashi et al., 2020). Since its first report in China in 2009 (Yu et al., 2011), the number of SFTS cases has continued to increase, making it an urgent focus in public health. It was also listed as a priority research disease by WHO (2017). According to the *National Guidelines for Diagnosis and Treatment of SFTS 2023 in China* published by the Health Commission of the People’s Republic of China (2023 guidelines) (NHC and PRC, 2023), SFTS is classified as mild, moderate, severe, and critical. The symptoms of critical SFTS include septic shock, multiple organ failure, and severe consciousness impairment. However, to date, there are no specific treatments for SFTS.

Stratifying patients upon admission to anticipate cases that may progress to critical or fatal SFTS is crucial for early intervention. Numerous studies have investigated the risk factors of SFTS. According to the 2023 guidelines, indicators of inflammation, hepatic, cardiac, renal, pancreatic, and coagulation functions are associated with the prognosis of SFTS (NHC and PRC, 2023). Among them, the ones that most strongly correlated with poor prognosis in SFTS included elevated levels of blood urea nitrogen (BUN), activated partial thromboplastin time (APTT), and lactate dehydrogenase (LDH) (Jia et al., 2017; Wang et al., 2019). However, research on the relationship between serum lipid profiles and SFTS prognosis is relatively limited.

Lipids play crucial roles in the progression of virus infections and cell-mediated immune responses. First, viruses remodel the host cell membrane, often taking advantage of the host’s lipid metabolic pathways to reshape the composition and structure of cell membranes. For example, most positive-stranded RNA viruses remodel the host cell membranes to create a favorable micro-environment for virus genomic RNA replication (Zhang et al., 2019). Second, viruses regulate host lipid metabolism. Most viruses modulate lipid synthesis, storage, and transport pathways in host cells to meet their own metabolic needs, which include changes in lipid droplet formation, fatty acid metabolism, and cholesterol biosynthesis (Girdhar et al., 2021). Third, lipid mediators, such as prostaglandins, leukotrienes, and sphingolipids, modulate host immune responses by regulating inflammation levels, immune cell recruitment, and cytokine production (Jaschonek and Muller, 1988; Van Hoecke et al., 2007; Ricciotti and FitzGerald, 2011). Viruses exploit these lipid mediators to evade host immune surveillance, promote immune evasion, or enhance viral pathogenesis. Finally, lipid rafts in the cell membrane participate in immune response. Lipid rafts are micro-domains on the cell membrane that contribute significantly to immune signaling and antigen presentation, influencing the activation and function of immune cells during viral infections (Roncato et al., 2022). However, the role of lipids in SFTSV infection remains unclear.

During the COVID-19 pandemic, many researchers analyzed the relationship between COVID-19 infection and aberrations in the host serum lipid profiles. They not only elucidated the role of cholesterol in the infection process (Sohrabi et al., 2021) but also discovered the benefits of statins for the treatment of COVID-19 (Zhang et al., 2020), thereby offering new insights for clinical therapy. However, the relationship between SFTS, a viral infectious disease that has been recognized for over a decade, and lipid metabolism remains unclear. Therefore, it is necessary to systematically evaluate the link between SFTS and the serum lipid profiles of patients.

In our previous lipidomics study on SFTS, we found significant dysregulation of lipid metabolism in SFTS (Supplementary Figure S1). This large-scale retrospective cohort study was designed to investigate the dysregulation in lipid metabolism further and understand the relationship between serum lipid profiles and SFTS prognosis. We believe our findings will provide new evidence for the treatment and prognosis of SFTS.

## 2 Materials and methods

### 2.1 Study design and participants

**Figure 1:**
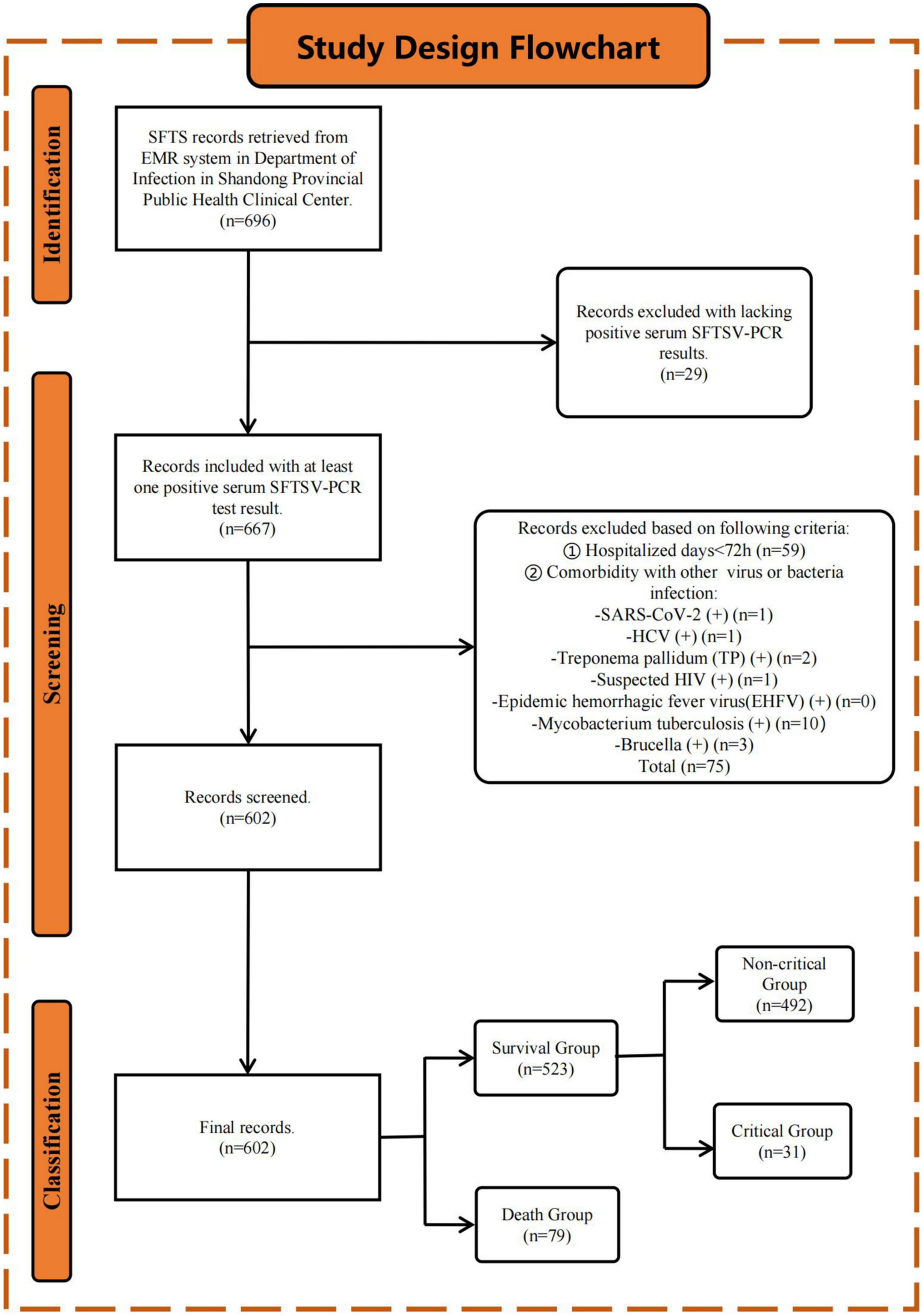
Study design flowchart. Initially, 696 patients diagnosed with SFTS were retrieved from the EMR system. After applying inclusion and exclusion criteria (see Method 2.1 section), 602 patients were finally included in the study. Patients were stratified into the S (*n* = 523) and D (*n* = 79) groups based on the endpoint events. The S group was further stratified into non-C (*n* = 492) and C (*n* = 31) groups based on clinical symptoms.

This retrospective study enrolled 696 hospitalized patients diagnosed with SFTS in the Department of Infectious Diseases at Shandong Public Health Clinical Center from January 2021 to December 2023. Inclusion Criteria: According to the diagnostic criteria of guidelines [edition 2010 (NHC and PRC, 2011) and edition 2023 (NHC and PRC, 2023)], published by the Health Commission of the People’s Republic of China, patients diagnosed with SFTS by clinical physicians with at least one positive serum SFTSV-PCR result were included. We excluded patients with (a) hospital stays of less than 72 h and (b) concomitant infections of severe acute respiratory syndrome coronavirus 2 (SARS-CoV-2), hepatitis C virus, Brucella, *Mycobacterium tuberculosis*, suspected human immunodeficiency virus (HIV), or syphilis that could affect our study results. Finally, 602 patients were included, and the detailed study design flowchart is shown in Figure 1.

Based on the endpoint events, patients were stratified into survival (S) and death (D) groups. The S group was further classified into non-critical (non-C) and critical (C) groups based on clinical symptoms. According to the 2023 guidelines, SFTS is classified as mild, moderate, severe, and critical based on the clinical symptoms. Patients with mild SFTS present with slight fatigue or discomfort, are self-limiting, and usually recover within a week. Moderate SFTS is characterized by notable general discomfort along with poor appetite, nausea, vomiting, and other gastrointestinal symptoms but no neurological symptoms. Patients with severe SFTS present with neurological symptoms such as drowsiness, confusion, stupor, pulmonary infections, and gastrointestinal bleeding. Patients with critical SFTS go into a coma (Glasgow coma scale ≤8), shock, or have other organ failures requiring ICU care and treatment. The non-C group in this study corresponds to mild, moderate, and severe SFTS, while the C group corresponds to critical SFTS. This study was approved by the Ethics Review Committee of Shandong Public Health Clinical Center. This study was conducted following the guidelines of the *Declaration of Helsinki of the World Medical Association*.

### 2.2 Clinical definitions

Co-infection with SARS-CoV-2 was defined as positive SARS-CoV-2 nucleic acid testing on admission. Co-infection with the hepatitis C virus was defined as positive hepatitis C virus antibody testing on admission. Co-infection with Brucella was defined as a positive standard tube agglutination test on admission. Co-infection with *Mycobacterium tuberculosis* was defined as positive T-SPOT testing on admission. Suspected HIV infection was defined as a positive HIV antibody test on admission. Co-infection with syphilis was defined as a positive *Treponema pallidum* particle agglutination (TPPA) test on admission. Body mass index (BMI) was calculated based on body height and weight. Chronic hepatitis B history was defined as positive hepatitis B virus surface antigen (HBsAg) testing on admission. Suspected hypothyroidism was defined as a serum thyroid stimulating hormone (TSH) level above the upper limit on admission. Taking statin medication within 1 month before admission was defined as statin history. Time from onset to admission was defined as the days from when patients first reported discomfort to the day of admission.

### 2.3 Data collection

Demographic information, personal and medical history, clinical features, and laboratory test results were extracted from electronic medical records (EMRs) using a standardized data collection method. Laboratory test results were collected from the first post-fasting sample obtained within 24 h of hospital admission. All patients were followed up for at least 28 days after admission. All laboratory test results were obtained from the clinical laboratory at Shandong Public Health Clinical Center. The data were carefully reviewed and confirmed by an experienced team of physicians and were checked twice to ensure accuracy.

According to the 2023 guidelines, 16 laboratory markers were identified that correlated with the prognosis of SFTS. These markers included inflammation indicators such as white blood cell (WBC) count, neutrophil (NEU) count, lymphocyte (LYM) count, C-reactive protein (CRP), and interleukin-6 (IL-6); hepatic function indicators such as alanine transaminase (ALT), aspartate transaminase (AST); cardiac function indicators including creatine kinase (CK) and LDH; renal function indicators including serum creatinine (Scr); pancreatic function indicators such as serum amylase (AMY) and serum lipase; and coagulation function indicators such as platelet (PLT) count, thrombin time (TT), APTT, and D-dimer. Additionally, serum lipid profiles were also collected, including levels of triglycerides (TGs), total cholesterol (TC), HDL-C, and low-density lipoprotein cholesterol (LDL-C). The units and normal ranges for these indicators are listed in Supplementary Table S4. All confounders and mediators are illustrated using directed acyclic graphs (DAGs) (Supplementary Figure S2).

### 2.4 Study size

To achieve 90% power to reject the null hypothesis of zero effect size when the population effect size is 0.5 and the significance level (alpha) is 0.05 using a two-sided two-sample equal-variance *t*-test (Cohen, 2013), the S and D groups required a minimum of 323 and 49 subjects, respectively. However, the actual number of samples in the study far exceeded the minimum required to achieve a moderate effect size calculation. Therefore, the conclusions are robust. The analyses were performed using *PASS* (version 2021).

### 2.5 Missing data imputation

To address missing data in demographic information and laboratory test results, the random forest model was utilized for missing value imputation based on the missForest package in R (Stekhoven and Bühlmann, 2012). The imputation results were reported based on the out-of-bag error rate of the random forest.

### 2.6 Propensity score matching

In the initial comparison of serum lipid profiles between the S and D groups, as well as between the non-C and C groups, propensity score matching (PSM) was used to adjust confounders. In this study, 13 suspected confounders were included, namely age, gender, BMI, time from onset to admission, medical history (hypertension history, diabetes history, cardiovascular disease history, cerebrovascular disease history, and chronic hepatitis B history), suspected hypothyroidism, personal history (smoking and drinking), and statin medication history. PSM was conducted using the MatchIt package in R, with a matching ratio of 4:1 for the S vs. D groups and non-C vs. C groups, without setting a caliper for matching. Other settings followed the default specifications of the program, including using the nearest neighbor matching method, propensity score calculation using logistic regression, and estimation of the average treatment effect on the treated (ATT). The standardized mean difference (SMD) before and after matching was calculated using the cobalt package, with an SMD of <0.1 indicating a balance between the two groups of variables.

### 2.7 Sensitivity analysis

The sensitivity analyses included subgroup analyses within different populations, treating LDL-C as both continuous and categorical type in multivariable regression, adjusting confounders using both multivariable regression and PSM, and using both logistic regression and Cox regression to analyze the relationship between LDL-C and SFTS prognosis.

### 2.8 Mediation analysis

Sixteen indicators were considered potential mediator variables based on their established association with adverse SFTS prognosis. First, the correlation between LDL-C and these 16 suspected mediator variables was examined using Pearson correlation tests. Then, mediation analysis was performed after adjusting for confounders using the bruceR package, which is based on packages of mediation, interactions, and lavaan in R. The bootstrap method was used to test mediation effects with 1,000 iterations. The reported mediation effect coefficient was standardized and obtained after standardizing continuous variables. Bonferroni correction was applied to adjust for multiple mediation tests. The procedure of mediation analysis followed the *Guideline for Reporting Mediation Analysis of Randomized Trials and Observational Studies (AGReMA) Statement* (Lee et al., 2021).

### 2.9 Statistical methods

Continuous data following normal distribution are presented as mean ± standard deviation and analyzed using t-tests. Continuous data with non-normal distribution are presented as median (interquartile range) and analyzed using the Wilcoxon rank-sum tests. Categorical data have been presented as sample size (percentage) and analyzed using Fisher’s exact tests. When converting continuous variables into categorical variables, “()” indicates an open interval, while “[]” indicates a closed interval. Restricted cubic splines were generated using the rms package in R. Cox regression analyses were performed, and Kaplan–Meier curves were calculated using the survival package in R. Correlation analysis was conducted using Pearson’s test. All *p*-values <0.05 were considered significant. Throughout the study, p-values have been reported with three significant digits and denoted by asterisks as follows: *, *p* < 0.05; **, *p* < 0.01; ***, *p* < 0.001; ****, *p* < 0.0001. All analyses were performed using R (version 4.3.1).

## 3 Results

### 3.1 Baseline data and clinical characteristics of patients with SFTS

The baseline characteristics of the study population are summarized in Table 1. We included 602 patients with SFTS in this study. Of them, 306 (50.83%) were men with a median age of 66.0 (58.0–72.0) years, median BMI of 22.86 (20.76–24.97), and median hospital stay of 10.0 (7.0–14.0) days.

**Table 1:** Baseline characteristics of SFTS patients.

**Variables**	**Total (*N* = 602)**	**Survival Group (*N* = 523)**	**Death Group (*N* = 79)**	***p*-value**	**Non-critical Group (*N* = 492)**	**Critical Group (*N* = 31)**	***p*-value**
**Clinical characteristics on admission**							
Gender				0.070			>0.999
Male	306 (50.83%)	258 (49.33%)	48 (60.76%)		243 (49.39%)	15 (48.39%)	
Female	296 (49.17%)	265 (50.67%)	31 (39.24%)		249 (50.61%)	16 (51.61%)	
Age	66.0 (58.0–72.0)	66.0 (57.0–72.0)	69.0 (66.0–74.0)	<0.001****	65.5 (57.0–72.0)	68.0 (61.5–73.5)	0.177
BMI (kg/m^2^)	22.86 (20.76–24.97)	22.89 (20.78–24.99)	22.60 (20.76–24.45)	0.464	22.93 (20.81–25.02)	22.22 (20.24–24.05)	0.106
Time from Onset to Admission (days)				0.686			0.648
≤4	143 (23.75%)	120 (22.94%)	23 (29.11%)	0.947	116 (23.58%)	4 (12.90%)	0.738
(4,6]	196 (32.56%)	171 (32.70%)	25 (31.65%)	>0.999	160 (32.52%)	11 (35.48%)	0.738
(6,8]	174 (28.90%)	151 (28.87%)	23 (29.11%)	>0.999	141 (28.66%)	10 (32.26%)	0.738
(8,10]	41 (6.81%)	37 (7.07%)	4 (5.06%)	>0.999	34 (6.91%)	3 (9.68%)	0.738
>10	48 (7.97%)	44 (8.41%)	4 (5.06%)	0.947	41 (8.33%)	3 (9.68%)	0.738
Length of Stay	10.0 (7.0–14.0)	11.0 (8.0–14.0)	6.0 (4.5–8.5)	<0.001****	10.0 (8.0–13.0)	34.0 (22.5–38.5)	<0.001****
**Past medical history**							
Hypertension History				0.119			>0.999
N	457 (75.91%)	403 (77.06%)	54 (68.35%)		379 (77.03%)	24 (77.42%)	
Y	145 (24.09%)	120 (22.94%)	25 (31.65%)		113 (22.97%)	7 (22.58%)	
Diabetes History				0.358			0.390
N	529 (87.87%)	462 (88.34%)	67 (84.81%)		436 (88.62%)	26 (83.87%)	
Y	73 (12.13%)	61 (11.66%)	12 (15.19%)		56 (11.38%)	5 (16.13%)	
Cardiovascular Disease History				0.813			>0.999
N	560 (93.02%)	487 (93.12%)	73 (92.41%)		458 (93.09%)	29 (93.55%)	
Y	42 (6.98%)	36 (6.88%)	6 (7.59%)		34 (6.91%)	2 (6.45%)	
Cerebrovascular Disease History				0.674			0.178
N	548 (91.03%)	477 (91.20%)	71 (89.87%)		451 (91.67%)	26 (83.87%)	
Y	54 (8.97%)	46 (8.80%)	8 (10.13%)		41 (8.33%)	5 (16.13%)	
Chronic Hepatitis B History				>0.999			0.357
N	578 (96.01%)	502 (95.98%)	76 (96.20%)		473 (96.14%)	29 (93.55%)	
Y	24 (3.99%)	21 (4.02%)	3 (3.80%)		19 (3.86%)	2 (6.45%)	
Suspected Hypothyroidism				0.160			>0.999
N	572 (95.02%)	494 (94.46%)	78 (98.73%)		464 (94.31%)	30 (96.77%)	
Y	30 (4.98%)	29 (5.54%)	1 (1.27%)		28 (5.69%)	1 (3.23%)	
Smoking History				0.632			0.211
N	499 (82.89%)	435 (83.17%)	64 (81.01%)		412 (83.74%)	23 (74.19%)	
Y	103 (17.11%)	88 (16.83%)	15 (18.99%)		80 (16.26%)	8 (25.81%)	
Drinking History				>0.999			>0.999
N	488 (81.06%)	424 (81.07%)	64 (81.01%)		399 (81.10%)	25 (80.65%)	
Y	114 (18.94%)	99 (18.93%)	15 (18.99%)		93 (18.90%)	6 (19.35%)	
Statin History				0.080			0.499
N	551 (91.53%)	483 (92.35%)	68 (86.08%)		453 (92.07%)	30 (96.77%)	
Y	51 (8.47%)	40 (7.65%)	11 (13.92%)		39 (7.93%)	1 (3.23%)	
**Laboratory test results**							
WBC	2.63 (1.71–4.70)	2.65 (1.76–4.88)	2.30 (1.56–4.04)	0.101	2.65 (1.75–4.88)	2.69 (1.90–4.49)	0.849
NEU	1.64 (1.05–3.30)	1.65 (1.05–3.30)	1.58 (1.09–3.30)	0.818	1.61 (1.03–3.30)	1.81 (1.32–3.50)	0.233
LYM	0.61 (0.40–1.04)	0.67 (0.40–1.10)	0.50 (0.31–0.78)	<0.001***	0.69 (0.40–1.10)	0.51 (0.37–0.74)	0.086
CPR	4.17 (1.50–10.40)	3.65 (1.34–9.50)	8.74 (3.89–16.28)	<0.001****	3.55 (1.25–8.96)	8.34 (3.54–17.59)	0.002**
IL-6	23.49 (7.82–68.32)	18.90 (6.66–48.29)	88.74 (45.12–230.51)	<0.001****	17.38 (6.32–41.52)	73.51 (31.31–115.84)	<0.001****
ALT	64.5 (38.0–127.8)	62.0 (36.0–123.5)	78.0 (45.5–166.0)	0.020*	59.0 (35.0–120.0)	115.0 (79.0–174.0)	<0.001****
AST	129.0 (72.0–290.5)	121.0 (68.0–244.0)	291.0 (134.5–496.5)	<0.001****	117.0 (64.8–228.8)	331.0 (212.0–560.5)	<0.001****
CK	370.5 (171.0–998.5)	330.0 (156.5–791.5)	1,003.0 (368.0–1,975.0)	<0.001****	310.0 (153.0–710.0)	1,244.0 (664.0–2,158.5)	<0.001****
LDH	574.0 (401.3–931.0)	544.0 (381.5–852.5)	1,002.0 (641.5–1,603.5)	<0.001****	518.5 (366.8–803.3)	1,217.0 (941.0–1,846.5)	<0.001****
Scr	66.00 (55.00–80.00)	65.00 (53.75–78.45)	77.00 (63.70–108.50)	<0.001****	64.85 (53.95–78.00)	70.00 (52.00–83.50)	0.202
AMY	87.0 (60.0–121.8)	85.0 (59.0–118.0)	111.0 (75.0–172.5)	<0.001****	83.0 (58.0–116.0)	118.0 (83.0–187.0)	<0.001***
Lipase	88.25 (48.53–162.20)	82.20 (46.45–147.45)	133.00 (74.05–252.70)	<0.001****	79.25 (45.95–142.60)	132.80 (77.25–231.85)	0.011*
PLT	58.0 (42.0–80.0)	61.0 (43.5–84.0)	45.0 (33.0–60.0)	<0.001****	62.5 (45.0–86.0)	40.0 (23.0–51.0)	<0.001****
TT	22.60 (20.30–25.30)	22.10 (20.10–24.95)	25.20 (23.10–30.05)	<0.001****	21.80 (20.10–24.50)	27.30 (24.60–31.60)	<0.001****
APTT	46.80 (40.10–55.80)	45.20 (39.30–52.15)	59.80 (52.15–69.25)	<0.001****	44.65 (39.10–51.30)	59.90 (50.10–66.70)	<0.001****
D-dimer	2.00 (1.01–4.50)	1.72 (0.94–3.68)	4.97 (2.90–8.00)	<0.001****	1.64 (0.90–3.06)	5.10 (2.71–7.72)	<0.001****
**Serum lipid profiles**							
TG	1.79 (1.22–2.56)	1.80 (1.23–2.57)	1.62 (1.22–2.28)	0.367	1.80 (1.20–2.53)	2.20 (1.61–3.26)	0.009**
Total Cholesterol	3.32 ± 0.86	3.35 ± 0.85	3.12 ± 0.86	0.032*	3.37 ± 0.85	3.07 ± 0.88	0.079
HDL-C	0.90 (0.75–1.07)	0.90 (0.76–1.06)	0.91 (0.67–1.18)	0.870	0.90 (0.76–1.06)	0.87 (0.75–0.97)	0.237
LDL-C	1.59 (1.22–2.01)	1.61 (1.28–2.09)	1.33 (1.01–1.70)	<0.001****	1.62 (1.30–2.11)	1.29 (0.95–1.67)	0.001**

Patients were stratified into S (*n* = 523) and D (*n* = 79) groups based on the endpoint events. Compared to the S group, patients in the D group were older [age: 69.0 (66.0–74.0) vs. 66.0 (57.0–72.0), *p* < 0.001], and had shorter hospital stays [6.0 (4.5–8.5) vs. 11.0 (8.0–14.0), *p* < 0.001)]. Significant differences were seen in the LYM, CRP, IL-6, ALT, AST, CK, LDH, Scr, AMY, lipase, PLT, TT, APTT, and D-dimer measures between the S and D groups. The D group, compared to the S group, had lower TC [3.12 ± 0.86 vs. 3.35 ± 0.85, *p* = 0.032] and lower LDL-C [1.33 (1.01–1.70) vs. 1.61 (1.28–2.09), *p* < 0.001]. Additionally, detailed information regarding the pre-admission use of statins in patients with SFTS is presented in Supplementary Table S1.

Within the S group, patients were further divided into non-C (*n* = 492) and C (*n* = 31) groups. In contrast to the S and D groups, no significant differences were seen in the age, LYM, Scr, or TC measures between the non-C and C groups. However, patients in the C group had higher TG levels compared to the non-C group [2.20 (1.61–3.26) vs. 1.80 (1.20–2.53), *p* = 0.009].

### 3.2 Lower serum LDL-C levels on admission are a potential poor prognostic indicator in SFTS

#### 3.2.1 Post-matching results

Seventy-nine patients from the D group were matched with 316 patients from the S group, and confounders were balanced after PSM (all SMD < 0.1). Post-matching results after adjusting the confounders using PSM showed that compared to the S group, patients in the D group had significantly lower LDL-C levels [1.33 (1.01–1.70) vs. 1.54 (1.19–1.99), *p* < 0.001, SMD = 0.590] (Table 2). Similarly, 31 patients from the C group were matched with 124 patients from the non-C group, and confounders were balanced after PSM (all SMD < 0.1). The post-matching results showed that compared to the non-C group, patients in the C group had significantly lower LDL-C levels [1.29 (0.95–1.67) vs. 1.61 (1.23–1.98), *p* = 0.007, SMD = 0.634], and significantly higher TG levels [2.20 (1.61–3.26) vs. 1.76 (1.23–2.43), *p* = 0.019, SMD = 0.408]. The lipid marker LDL-C showed the most significant difference (with the largest SMD value) between the S and D groups as well as the non-C and C groups.

**Table 2:** Post-matching lipid profile results of SFTS patients.

**Variable**	**Total (*N* = 395)**	**Survival Group (*N* = 316)**	**Death Group (*N* = 79)**	***p*-value**	**SMD**	**Total (*N* = 155)**	**Non-critical Group (*N* = 124)**	**Critical Group (*N* = 31)**	***p*-value**	**SMD**
**Confounders**										
Age	69.0 (64.0–74.0)	69.0 (64.0–74.0)	69.0 (66.0–74.0)	0.735	0.093	67.3 ± 9.2	67.4 ± 9.4	66.8 ± 8.4	0.750	0.066
Gender				0.704	0.025				0.841	0.032
Male	232 (58.73%)	184 (58.23%)	48 (60.76%)			71 (45.81%)	56 (45.16%)	15 (48.39%)		
Female	163 (41.27%)	132 (41.77%)	31 (39.24%)			84 (54.19%)	68 (54.84%)	16 (51.61%)		
BMI (kg/m^2^)	22.72 (20.76–24.73)	22.85 (20.76–24.76)	22.60 (20.76–24.45)	0.843	0.036	22.10 ± 3.35	22.11 ± 3.22	22.07 ± 3.89	0.960	0.010
Time from Onset to Admission (days)				0.925					0.830	
≤4	108 (27.34%)	85 (26.90%)	23 (29.11%)	>0.999	0.022	24 (15.48%)	20 (16.13%)	4 (12.90%)	>0.999	0.032
(4, 6]	127 (32.15%)	102 (32.28%)	25 (31.65%)	>0.999	0.006	56 (36.13%)	45 (36.29%)	11 (35.48%)	>0.999	0.008
(6, 8]	122 (30.89%)	99 (31.33%)	23 (29.11%)	>0.999	0.022	53 (34.19%)	43 (34.68%)	10 (32.26%)	>0.999	0.024
(8, 10]	23 (5.82%)	19 (6.01%)	4 (5.06%)	>0.999	0.009	13 (8.39%)	10 (8.06%)	3 (9.68%)	>0.999	0.016
>10	15 (3.80%)	11 (3.48%)	4 (5.06%)	>0.999	0.016	9 (5.81%)	6 (4.84%)	3 (9.68%)	>0.999	0.048
Hypertension History				0.582	0.032				>0.999	0.024
N	280 (70.89%)	226 (71.52%)	54 (68.35%)			117 (75.48%)	93 (75.00%)	24 (77.42%)		
Y	115 (29.11%)	90 (28.48%)	25 (31.65%)			38 (24.52%)	31 (25.00%)	7 (22.58%)		
Diabetes History				>0.999	0.000				>0.999	0.008
N	335 (84.81%)	268 (84.81%)	67 (84.81%)			131 (84.52%)	105 (84.68%)	26 (83.87%)		
Y	60 (15.19%)	48 (15.19%)	12 (15.19%)			24 (15.48%)	19 (15.32%)	5 (16.13%)		
Cardiovascular Disease History				>0.999	0.003				>0.999	0.016
N	366 (92.66%)	293 (92.72%)	73 (92.41%)			143 (92.26%)	114 (91.94%)	29 (93.55%)		
Y	29 (7.34%)	23 (7.28%)	6 (7.59%)			12 (7.74%)	10 (8.06%)	2 (6.45%)		
Cerebrovascular Disease History				>0.999	0.009				0.775	0.024
N	352 (89.11%)	281 (88.92%)	71 (89.87%)			133 (85.81%)	107 (86.29%)	26 (83.87%)		
Y	43 (10.89%)	35 (11.08%)	8 (10.13%)			22 (14.19%)	17 (13.71%)	5 (16.13%)		
Chronic Hepatitis B History				>0.999	0.003				0.628	0.024
N	379 (95.95%)	303 (95.89%)	76 (96.20%)			148 (95.48%)	119 (95.97%)	29 (93.55%)		
Y	16 (4.05%)	13 (4.11%)	3 (3.80%)			7 (4.52%)	5 (4.03%)	2 (6.45%)		
Suspected Hypothyroidism				>0.999	0.003				0.361	0.024
N	391 (98.99%)	313 (99.05%)	78 (98.73%)			153 (98.71%)	123 (99.19%)	30 (96.77%)		
Y	4 (1.01%)	3 (0.95%)	1 (1.27%)			2 (1.29%)	1 (0.81%)	1 (3.23%)		
Smoking History				>0.999	0.009				>0.999	0.008
N	317 (80.25%)	253 (80.06%)	64 (81.01%)			114 (73.55%)	91 (73.39%)	23 (74.19%)		
Y	78 (19.75%)	63 (19.94%)	15 (18.99%)			41 (26.45%)	33 (26.61%)	8 (25.81%)		
Drinking History				0.876	0.013				>0.999	0.000
N	316 (80.00%)	252 (79.75%)	64 (81.01%)			125 (80.65%)	100 (80.65%)	25 (80.65%)		
Y	79 (20.00%)	64 (20.25%)	15 (18.99%)			30 (19.35%)	24 (19.35%)	6 (19.35%)		
Statin History				0.568	0.022				>0.999	0.008
N	347 (87.85%)	279 (88.29%)	68 (86.08%)			151 (97.42%)	121 (97.58%)	30 (96.77%)		
Y	48 (12.15%)	37 (11.71%)	11 (13.92%)			4 (2.58%)	3 (2.42%)	1 (3.23%)		
**Serum lipid profiles**										
TG	1.68 (1.22–2.38)	1.69 (1.22–2.38)	1.62 (1.22–2.28)	0.707	0.056	1.82 (1.31–2.75)	1.76 (1.23–2.43)	2.20 (1.61–3.26)	0.019*	0.408
Total Cholesterol	3.29 ± 0.86	3.33 ± 0.86	3.13 ± 0.86	0.062	0.237	3.28 ± 0.83	3.34 ± 0.81	3.07 ± 0.88	0.139	0.298
HDL-C	0.90 (0.74–1.10)	0.90 (0.76–1.08)	0.91 (0.67–1.18)	0.839	0.007	0.87 (0.77–1.06)	0.88 (0.77–1.07)	0.87 (0.75–0.97)	0.334	0.185
LDL-C	1.51 (1.16–1.96)	1.54 (1.19–1.99)	1.33 (1.01–1.70)	<0.001 ***	0.590	1.57 (1.18–1.93)	1.61 (1.23–1.98)	1.29 (0.95–1.67)	0.007**	0.634

#### 3.2.2 Logistic regression analysis

Three different logistic regression models were separately applied to assess the relationship between LDL-C and poor prognosis in SFTS: the unadjusted logistic regression (crude model), logistic regression adjusted for confounders (adjusted model), and logistic regression with post-matching data (after PSM model) (Table 3). Comparing the results between the S and D groups, lower LDL-C levels were found to be associated with mortality [crude model, OR = 0.352 (0.217–0.554), *p* < 0.001; adjusted model, OR = 0.393 (0.234–0.638), *p* < 0.001; after PSM model, OR = 0.425 (0.260–0.671), *p* < 0.001]. Comparisons between the non-C and C groups yielded similar results [crude model, OR = 0.316 (0.148–0.632), *p* = 0.002; adjusted model, OR = 0.249 (0.105–0.544), *p* < 0.001; after PSM model, OR = 0.328 (0.136–0.714), *p* = 0.008]. Furthermore, for sensitivity analysis, we treated LDL-C as a categorical variable (based on quantiles) and observed similar trends across all models.

**Table 3:** Logistic regression model between LDL-C and poor prognosis of SFTS.

**Crude Model**		**Adjusted Model**		**After PSM Model**	
**Variables**	**OR**	**Variables**	**OR**	**Variables**	**OR**
**Survival group vs. Death group**					
LDL-C	0.352 (0.217–0.554)		0.393 (0.234–0.638)		0.425 (0.260–0.671)
*p*-value	<0.001****		<0.001***		<0.001***
**Quantile:**					
Q1 [0.14, 1.22] 0.98	Ref	Q1 [0.14,1.22] 0.98	Ref	Q1 [0.14,1.16] 0.95	Ref
Q2 (1.22, 1.59] 1.42	0.542 (0.291–0.988)	Q2 (1.22,1.59] 1.42	0.573 (0.299–1.077)	Q2 (1.16,1.51] 1.35	0.739 (0.386–1.397)
Q3 (1.59, 2.01] 1.78	0.539 (0.287–0.991)	Q3 (1.59,2.01] 1.78	0.588 (0.301–1.123)	Q3 (1.51,1.96] 1.70	0.567 (0.287–1.097)
Q4 (2.01, 4.55] 2.38	0.175 (0.069–0.388)	Q4 (2.01,4.55] 2.38	0.211 (0.081–0.489)	Q4 (1.96,4.38] 2.35	0.300 (0.131–0.638)
*p*-value for trend	<0.001****		<0.001***		0.002**
**Non-critical group vs. Critical group**					
LDL-C	0.316 (0.148–0.632)		0.249 (0.105–0.544)		0.328 (0.136–0.714)
P-value	0.002**		<0.001***		0.008**
**Quantile:**					
Q1 [0.14, 1.28] 1.02	Ref	Q1 [0.14,1.28] 1.02	Ref	Q1 [0.50,1.18] 0.94	Ref
Q2 (1.28, 1.61] 1.46	0.384 (0.133–0.979)	Q2 (1.28,1.61] 1.46	0.357 (0.117–0.976)	Q2 (1.18,1.57] 1.37	0.348 (0.110–0.997)
Q3 (1.61,2.09] 1.83	0.441 (0.163–1.083)	Q3 (1.61,2.09] 1.83	0.407 (0.142–1.063)	Q3 (1.57,1.92] 1.70	0.419 (0.140–1.165)
Q4 (2.09, 4.55] 2.45	0.187 (0.043–0.585)	Q4 (2.09,4.55] 2.45	0.147 (0.031–0.500)	Q4 (1.92,3.69] 2.33	0.212 (0.055–0.670)
*p*-value for trend	0.006**		0.004**		0.014*

#### 3.2.3 Restricted cubic spline results

**Figure 2:**
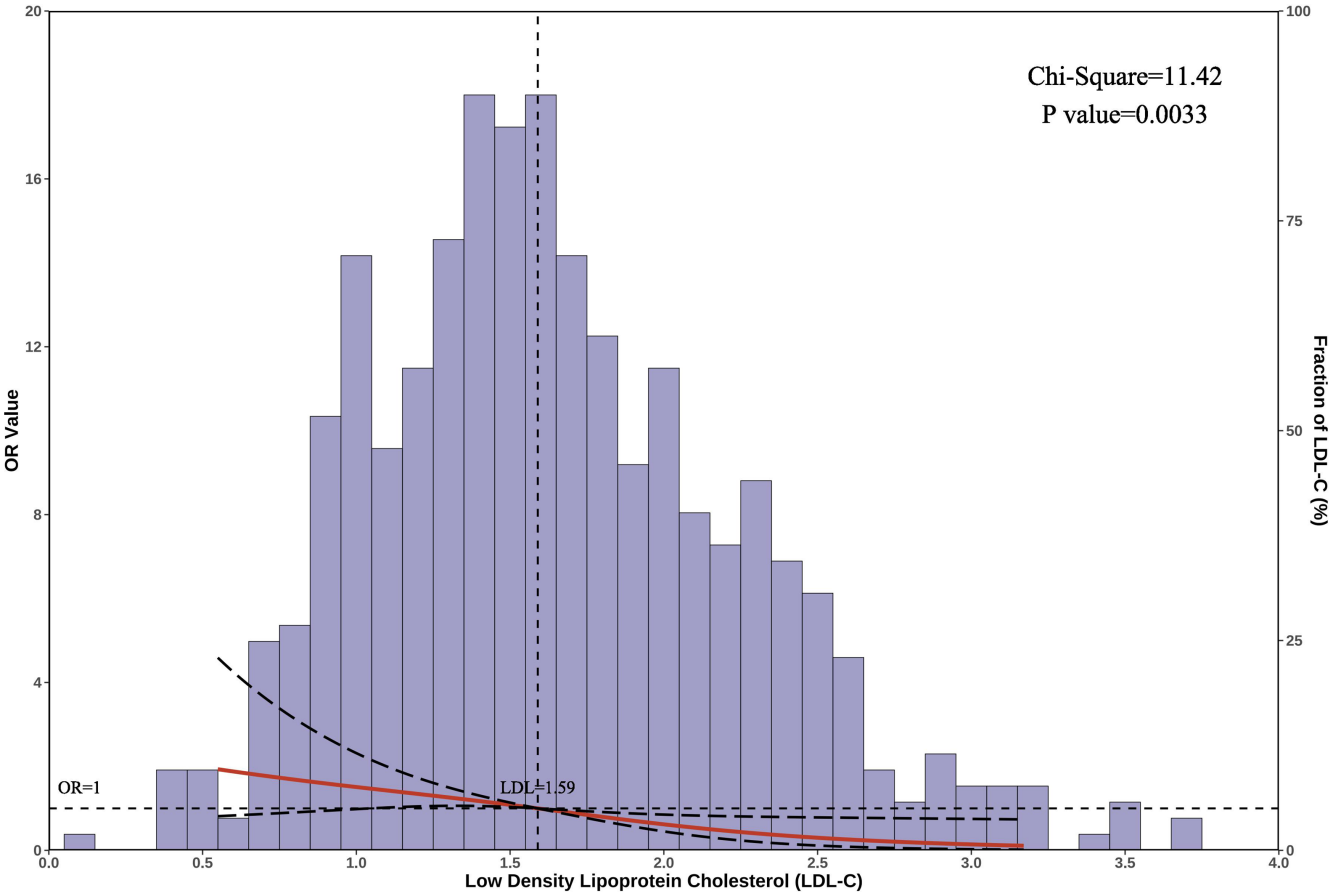
Restricted cubic spline analysis. The graph displays the correlation between serum LDL-C levels and the risk of mortality in patients with SFTS. The horizontal axis represents the serum LDL-C levels on admission, the left vertical axis shows the odds ratio (OR) values, and the right vertical axis represents the proportion of patients with each LDL-C level in the total population. The cutoff LDL-C value is 1.59 mmol/L. After adjusting for confounders, the chi-square value for the overall model was 11.42, with a *p*-value of 0.003.

After adjusting for confounders, the restricted cubic spline results revealed a unidirectional relationship between LDL-C levels and SFTS mortality risk (*p* = 0.003) (Figure 2). The cutoff value for LDL-C was 1.59 mmol/L (at which point OR = 1), allowing for the categorization of LDL-C into low LDL-C (≤ 1.59 mmoL/L) and high LDL-C (> 1.59 mmol/L) groups. Hence, for patients with serum LDL-C ≤ 1.59 mmol/L on admission, heightened vigilance by clinicians is warranted.

#### 3.2.4 Survival analysis

**Figure 3:**
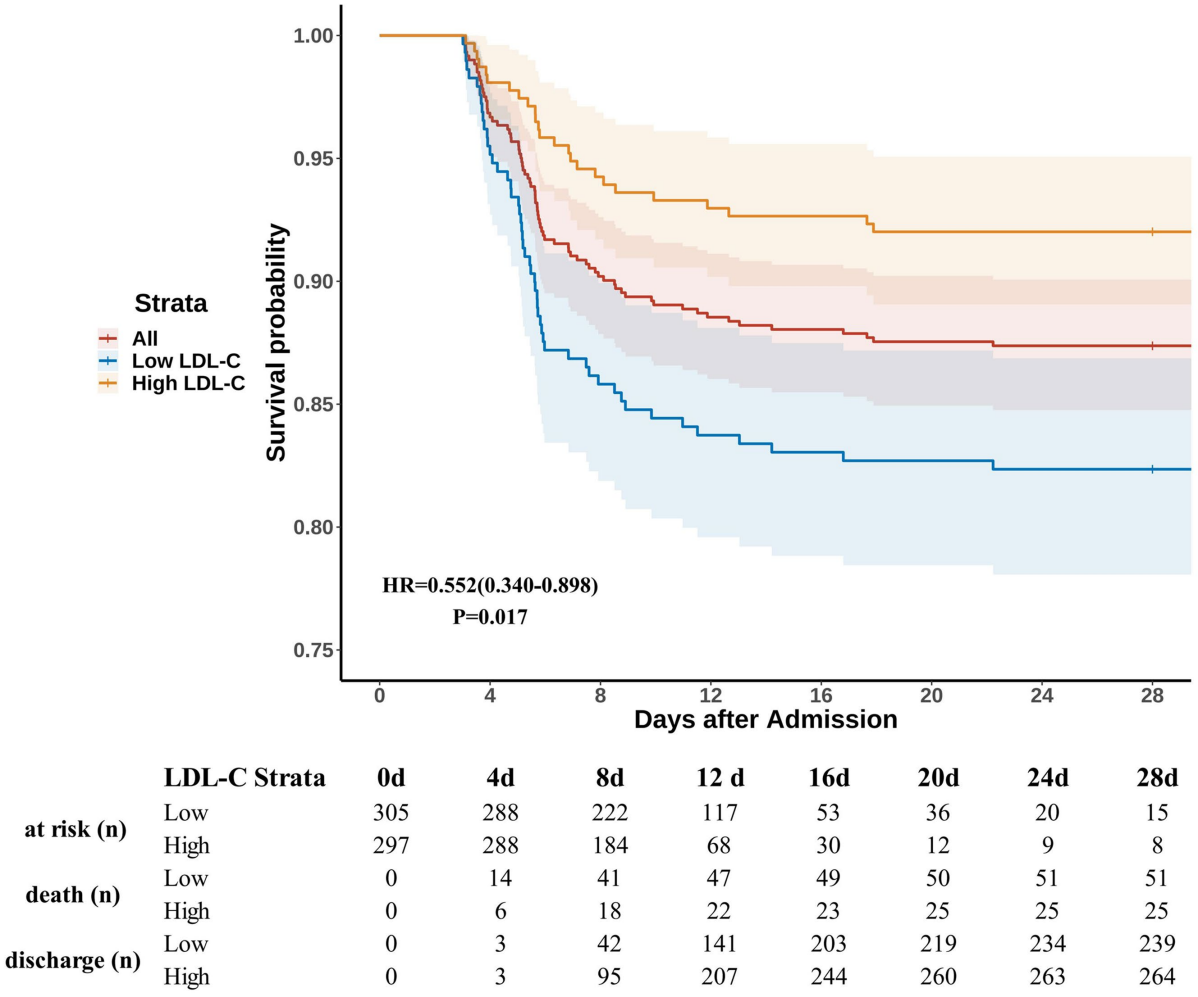
Survival curves. Patients were followed after admission for at least 28 days. LDL-C levels of ≤1.59 mmol/L and > 1.59 mmol/L were defined as low LDL-C and high LDL-C, respectively. HR and *p*-values were calculated based on Cox proportional hazards regression after adjusting for confounders. The shaded areas represent the 95% confidence intervals. The table below the figure shows the number of patients at risk, deaths, and discharges at each specified time point. The number “at risk” is defined as the total number minus the number of deaths and discharges.

All patients were followed for at least 28 days from admission. The Kaplan–Meier curves indicated higher and earlier mortality rates within the first week post-admission in the low-LDL-C group compared to the high-LDL-C group (Figure 3). After adjusting for confounders using the Cox proportional hazards regression, the low-LDL-C group exhibited a higher mortality risk compared to the high-LDL-C group [HR = 0.552 (0.340–0.898), *p* = 0.017].

#### 3.2.5 Subgroup analysis and the degree of interference of confounders

As a part of the sensitivity analysis, we investigated the relationship between LDL-C levels and the risk of death in different subgroups (Supplementary Figure S3). The subgroup analysis indicated a relationship between low LDL-C levels and poor prognosis in most subgroups. However, in subgroups characterized by age > 75, BMI < 18.5, time from onset to admission >13 days, and history of diabetes, cardiac disease, cerebrovascular disease, chronic hepatitis B, suspected hypothyroidism, smoking, drinking, and statin medication, the relationship between LDL-C and SFTS prognosis was no longer significant (*p* < 0.05). There were no significant interaction effects between LDL-C and any confounders (all *p for interaction > 0.05*).

### 3.3 Gradual increase in LDL-C levels is associated with a favorable prognosis in SFTS

**Figure 4:**
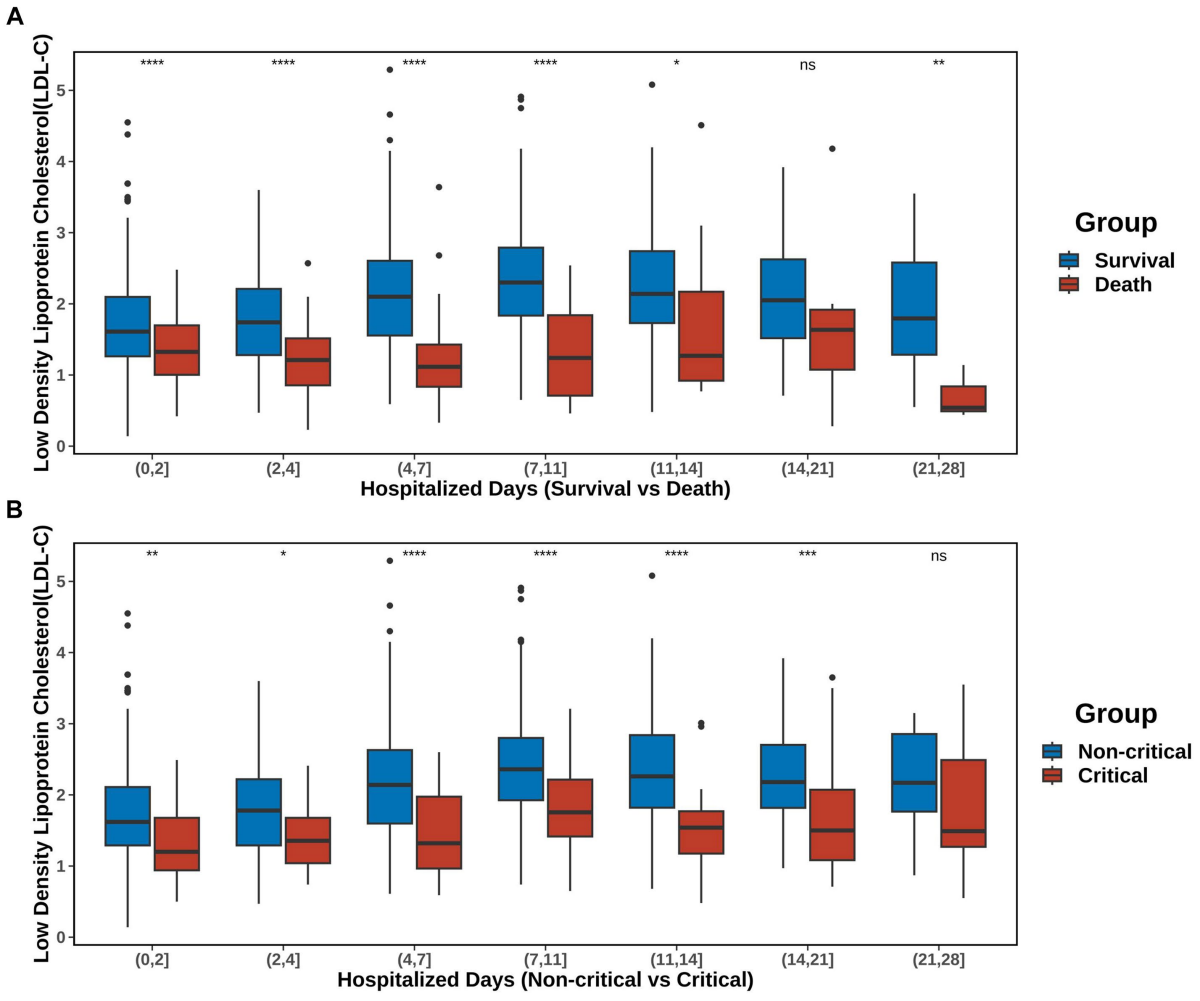
Trends in LDL-C over time. The post-admission period was divided into seven intervals: (0, 2), (2, 4), (4, 7), (7, 11), (11, 14), (14, 21), and (21, 28) days. **(A)** Changes in LDL-C levels in the S and D groups. **(B)** Changes in the serum LDL-C levels in the non-C and C groups. Detailed data are presented in Supplementary Table S2. The significance of *p*-values is denoted as follows: not significant *p* ≥ 0.05; *p < 0.05; **p < 0.01; ***p < 0.001; ****p < 0.0001.

The trend of serum LDL-C levels over time within 28 days after admission is shown in Figure 4 and Supplementary Table S2. The post-admission period was divided into seven intervals: (0, 2], (2, 4], (4, 7], (7, 11], (11, 14], (14, 21], and (21, 28] days. Comparison between S vs. D groups showed that, except for the period of (14, 21], the LDL-C levels in the D group were consistently lower than those in the S group. Furthermore, from admission to day 11, the serum LDL-C levels in the S group gradually increased. Comparison between non-C vs. C groups showed that, except for the period of (21, 28], the LDL-C levels in the C group were consistently lower than those in the non-C group. Similarly, from admission to day 11, the serum LDL-C levels in the non-C group gradually increased. However, the same trend was not observed in patients from the D and C groups.

### 3.4 Low LDL-C levels reflect poor hepatic function, cardiac function, and coagulation function in patients with SFTS

The analyses described above suggested that lower LDL-C levels may be associated with poor prognosis in SFTS. However, the mechanisms underlying the relationship between low LDL-C levels and adverse SFTS outcomes remain unclear. Sixteen indicators associated with adverse SFTS outcomes were considered as potential intermediary variables (Supplementary Figure S2). After excluding variables that were not significantly correlated with LDL-C using the Pearson correlation analysis, we found significant associations between LDL-C and all 15 of the indicators except for CRP (Supplementary Figure S4).

**Figure 5:**
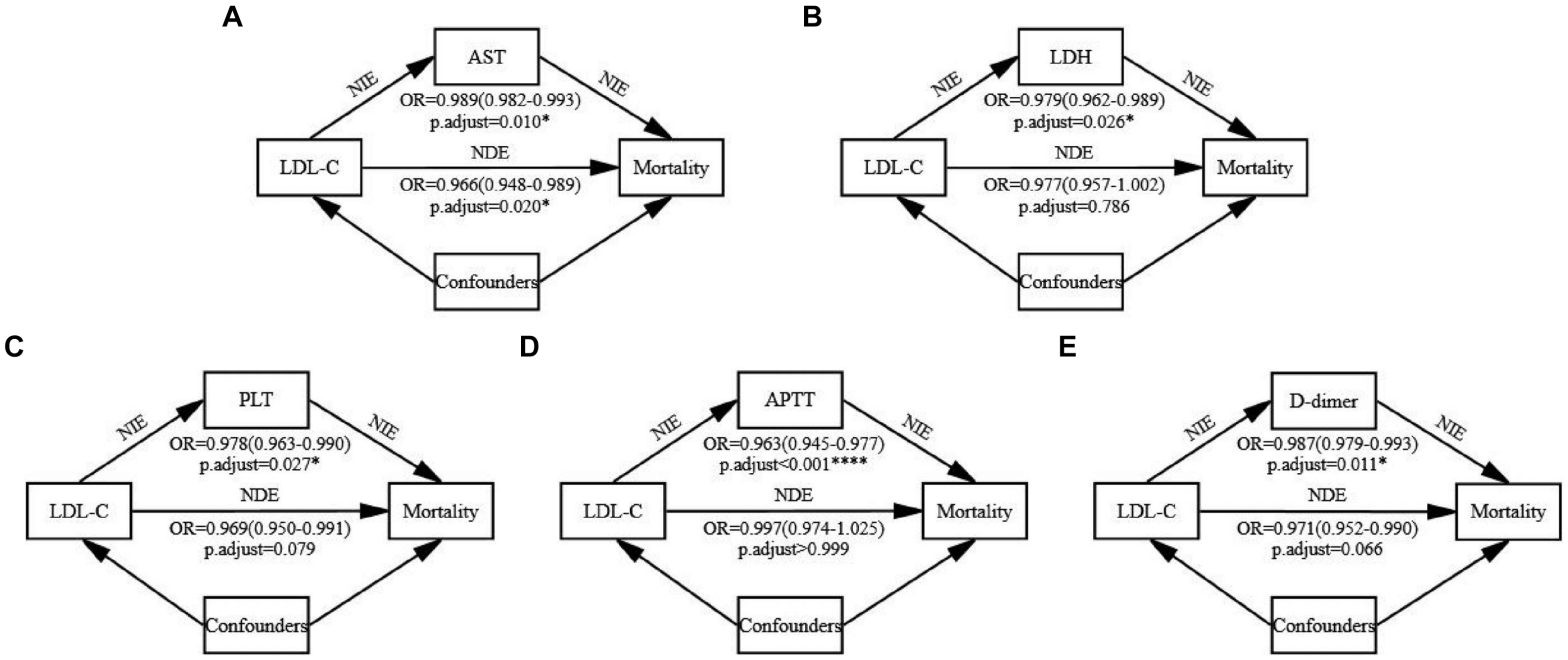
Mediation analysis. The mediation effects of the five significant indicators were examined using the bootstrap method with 1,000 iterations after adjusting for confounders. All continuous variables were standardized before the analysis, and the standardized coefficients have been reported. The *p*-values were adjusted using the Bonferroni method and reported as *p*-adjust values. **(A)** The mediating process of LDL-C → AST → Mortality. **(B)** The mediating process of LDL-C → LDH → Mortality. **(C)** The mediating process of LDL-C → PLT → Mortality. **(D)** The mediating process of LDL-C → APTT → Mortality. **(E)** The mediating process of LDL-C → D-dimer → Mortality. Detailed data are presented in Supplementary Table S3. NIE, natural indirect effect. NDE, natural direct effect.

After excluding CRP, mediation analysis was conducted on the remaining 15 potential indicators after adjusting for confounders in patients from *S* vs. *D* groups (Figure 5, Supplementary Figure S5, Supplementary Table S3). Significant mediation effects were observed in five indicators: AST, LDH, PLT, APTT, and D-dimer (all natural indirect effect (NIE) *p.adjust* < 0.05). These variables represented hepatic (AST), cardiac (LDH), and coagulation (PLT, APTT, and D-dimer) functions. Among them, low LDL-C → AST → mortality is a partial mediation process (natural direct effect (NDE) *p.adjust =* 0.020), while the others were complete mediation processes (NDE *p.adjust* > 0.05). The mediation analysis revealed that lower LDL-C levels may reflect poorer hepatic, cardiac, and coagulation functions, thereby resulting in higher mortality.

## 4 Discussion

Our study included 602 patients with SFTS and follow-ups exceeding 28 days. We observed significantly lower serum LDL-C levels in the D and C groups compared to the S and non-C groups. This finding persisted after adjusting for confounders using PSM. Further analysis using the crude, adjusted, and after PSM models consistently indicated that low LDL-C levels may be associated with poorer prognosis in SFTS. Restrictive cubic spline analysis revealed a unidirectional relationship between LDL-C levels and mortality risk, with a cutoff value of 1.59 mmol/L. In patients stratified based on LDL-C levels into low and high groups, the Kaplan–Meier curves and Cox proportional hazard regression model showed higher and earlier mortality rates in the low-LDL-C group. The subgroup analysis showed the degree of interference of confounders on the association between LDL-C levels and SFTS prognosis. The analysis of LDL-C trends over time revealed a gradual increase in serum LDL-C levels with improving clinical conditions in the S and non-C groups. Finally, the mediation analysis suggested that low LDL-C levels may result in increased mortality in patients with SFTS by affecting their hepatic, cardiac, and coagulation functions.

Previous studies have reported an association between viral infections and dyslipidemia, with significant alterations in serum lipid profiles in infections including HIV (Kirchner, 2012), dengue virus (Lima et al., 2019), SARS-CoV-2 (Aparisi et al., 2021), among others. Particularly, multiple studies on SARS-CoV-2 have reported the correlations between low LDL-C and HDL-C levels and poor prognoses (Aparisi et al., 2021; Chidambaram et al., 2022; Feingold, 2023). Additionally, a retrospective cohort study indicated an association between low LDL-C levels and increased mortality in infections (Kaysen et al., 2018), while another cohort study found a significant correlation between low LDL-C levels and higher long-term incidence of community-acquired sepsis (Guirgis et al., 2016). However, current research on the association between serum lipids and SFTS prognosis is limited. We only identified two studies on the relationship between blood lipid profiles and SFTS prognosis. In a study using machine learning methods, Zheng et al. reported that serum cholesterol may be a relevant indicator of mortality in patients with SFTS (Zheng et al., 2023). Huang et al. suggested an association between low serum HDL-C levels and adverse outcomes in SFTS (Huang et al., 2023). However, our findings suggest a relationship between low LDL-C levels and poor prognosis in SFTS. Differences in study populations, designs, and methodologies may lead to differing conclusions. Thus, further research is needed to confirm the association between serum lipid profiles and SFTS prognosis.

Our study provides a clinically relevant cutoff value for serum LDL-C levels (1.59 mmol/L). For patients with SFTS whose serum LDL-C levels are below 1.59 mmol/L, clinical attention should be heightened, as this may signify a poor prognosis. However, this value is not absolute, as indicated by the results of the subgroup analysis. In patients who are elderly (>75 years), have a low BMI (<18.5), have time from onset to admission >13 days, and have a history of diabetes, cardiac disease, cerebrovascular disease, chronic hepatitis B, smoking, drinking, or statin medication, these confounders may influence the LDL-C levels, which in turn may not accurately reflect SFTS prognosis. Additionally, in the subgroup with time from onset to admission within (7, 13] days, the correlation between LDL-C level and mortality reached its highest [OR = 0.060 (0.008–0.287), *p* = 0.002], indicating a significant decrease in LDL-C levels among patients in the D group during this period. Typically, SFTS progresses through three distinct stages: the fever stage (3–7 days after symptoms onset), the multiple organ dysfunction (MOD) stage (7–13 days after symptoms onset), and the convalescence stage (11–19 days after symptoms onset) (Gai et al., 2012). The highest correlation in the (7, 13] days subgroup indicates that the decrease in LDL-C levels is particularly significant during the MOD stage, where mortality also peaks. This also supports the notion that low LDL-C levels can reflect the disease status in patients with SFTS. In contrast, the correlation between LDL-C levels and mortality disappears in the subgroup with time from onset to admission >13 days. A possible reason is that the majority of deceased individuals died within 2 weeks after symptom onset, leaving very few patients who survived without receiving specialized treatment within 13 days after symptom onset.

Finally, mediation analysis was conducted to explore why lower levels of LDL-C are associated with an increased mortality risk in SFTS. We found that LDL-C may mediate mortality through five indicators: AST, LDH, PLT, APTT, and D-dimer. While the mediation through AST was partial, it was full through the other four indicators, pointing to some overlap in the meaning between LDL-C and AST. AST is an indicator of hepatic function, suggesting that while LDL-C levels can directly reflect the hepatic function level, they can only indirectly reflect the cardiac and coagulation functions. This is reasonable, as the relationship between LDL-C and liver function is significant, with multiple studies reporting an association between low serum LDL-C levels and impaired hepatic function (Ghadir et al., 2010; Chrostek et al., 2014; Jiang et al., 2014). However, it is important to note that the mediation effect between LDL-C and cardiac and coagulation functions only demonstrates a significant correlation between these parameters but does not prove a causal relationship. Further studies are required to confirm causality between them.

This study included a relatively large number of patients with SFTS, strengthening our ability to control and analyze the confounding variables. However, our study still has some limitations. First, despite correcting for the potential effects of numerous confounders, unquantifiable confounders may still exist. For instance, SFTS predominantly affects farmers, suggesting that the patient’s socioeconomic status could be a potential confounding factor. Unfortunately, many of the deceased patients had voluntarily abandoned treatment due to economic reasons, and some of them could have survived with adequate treatment. Thus, these potential confounders that were not included in the analysis may have introduced some degree of bias in the conclusions. Second, the retrospective study was limited to cases from the Shandong Public Health Clinical Center. Therefore, the findings may not apply to patients from other regions. Third, in some subgroup analyses, the small sample size in the D group may have led to unstable conclusions, thus requiring cautious interpretation. Finally, the analysis of mediation effects is more theoretical than statistical. We selected 16 potential mediator variables based on existing evidence, but they may not encompass all the actual mediators. While the PROCESS model (Hayes, 2012) included 76 pre-defined models of mediation and moderation effects, we only utilized the simplest mediation analysis and did not consider more complex models. However, in any case, our analysis indicates a relationship between low LDL-C levels and poor prognosis in SFTS, providing some theoretical explanation based on mediation effects.

## 5 Conclusion

Lower LDL-C levels on admission may be associated with poor prognosis in SFTS. Patients with LDL-C levels below 1.59 mmol/L should be closely monitored, as they may have a poor prognosis. Finally, mediation analysis revealed that lower LDL-C levels may mediate mortality through poor hepatic, cardiac, and coagulation functions in patients with SFTS.

## Data availability statement

The raw data supporting the conclusions of this article will be made available by the authors, without undue reservation.

## Ethics statement

The studies involving humans were approved by Ethics Review Committee of Shandong Public Health Clinical Center [GWLCZXEC2024-44-1]. The studies were conducted in accordance with the local legislation and institutional requirements. Written informed consent for participation was not required from the participants or the participants’ legal guardians/next of kin because This is a retrospective study that requires only data recorded in the hospital case system, does not intervene on the patients, does not cause any additional invasions or risks to the patients, and meets the criteria for free informed consent.

## Author contributions

SG: Formal analysis, Investigation, Methodology, Visualization, Writing – original draft, Writing – review & editing. QD: Investigation, Writing – original draft. MMZ: Conceptualization, Investigation, Writing – original draft. LRT: Project administration, Supervision, Writing – review & editing, Resources. YJY: Conceptualization, Data curation, Supervision, Writing – original draft. SGG: Funding acquisition, Project administration, Supervision, Writing – review & editing.
