# Supplementary material for: Lower serum LDL-C levels are associated with poor prognosis in severe fever with thrombocytopenia syndrome: a single-center retrospective cohort study
Source: Front Microbiol. 2024 Jun 24;15:1412263. doi: 10.3389/fmicb.2024.1412263 (PMC11229679; doi:10.3389/fmicb.2024.1412263)
Supplement: Supplementary file 1 [file Data_Sheet_1.pdf]

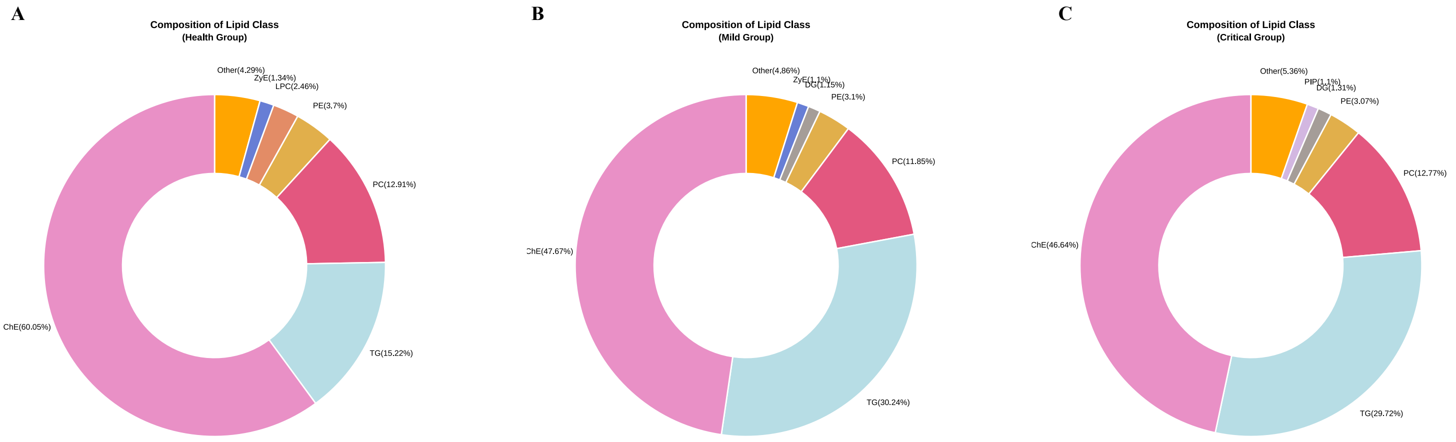

Figure S1. Analysis of Serum Lipidomic Subclass Content. (A) Composition of lipidome subclasses in the healthy group. (B) Composition of lipidome subclasses in the mild group. (C) Composition of lipidome subclasses in the critical group. Compared to the healthy group, SFTS patients exhibit significantly decreased ChE and increased TG contents. Note: These results are based on our previous lipidomics study on SFTS patients. The "mild" group corresponds to the "Non-C" group in this study, and the "C" group corresponds to the combined "critical" and "death" groups in this study. ChE, cholesterol ester; TG, triglycerides; PC, phosphatidylcholine; PE, phosphatidylethanolamine; LPC, lysophosphatidylcholine; DG, diacylglycerol; ZyE, zymosterol; Other, other lipid subclasses that not displayed.

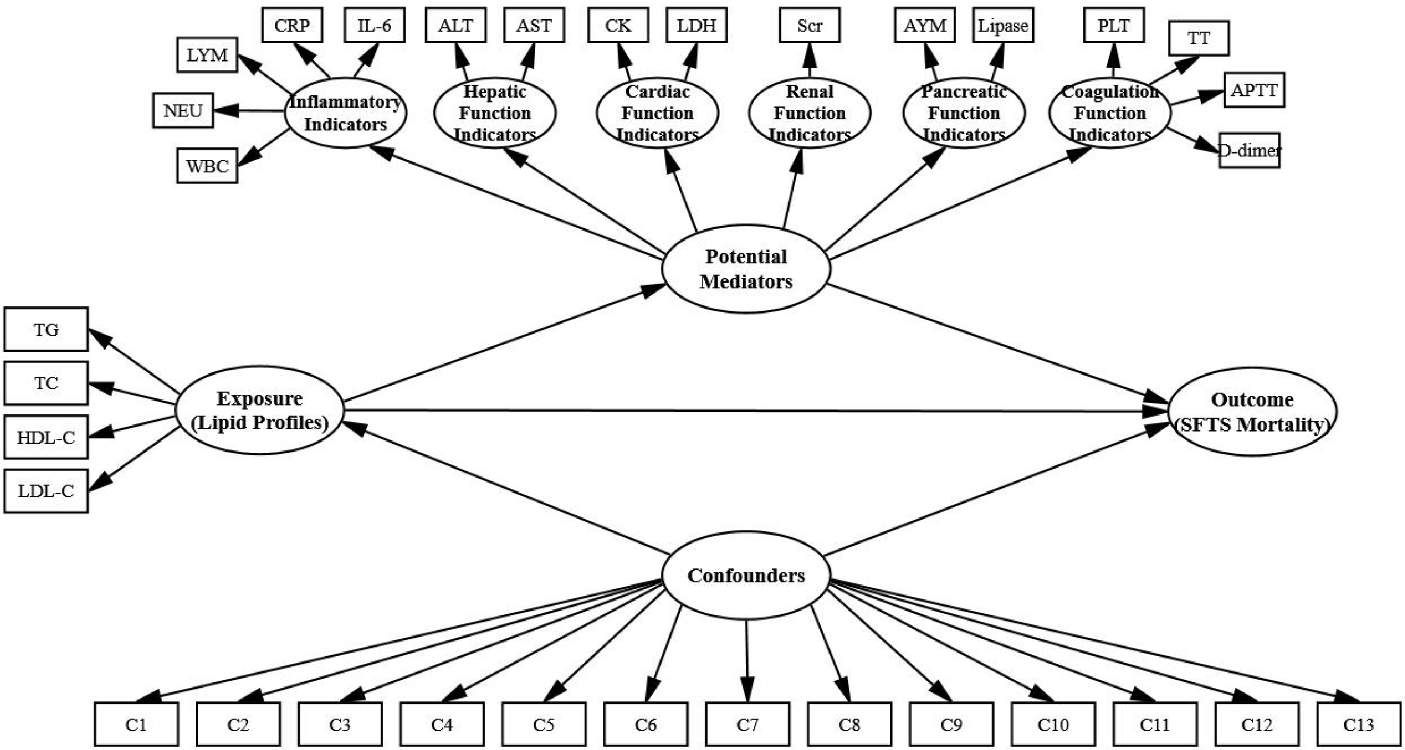

Figure S2. Directed Acyclic Graph (DAG). The directed acyclic graph illustrates the confounders and potential mediators considered in this study. C1 to C13 represent 13 confounding factors, respectively, including gender, age, body mass index (BMI), history of hypertension, diabetes, cerebrovascular disease, cardiovascular disease, chronic hepatitis B, suspected hypothyroidism, statin use, smoking, drinking, and time from onset to admission.

Variables	N	Survival Mean	Survival SD	N	Death Mean	Death SD	OR[95%CI]	P value	P for Interaction
**all**	523	1.70	0.63	79	1.37	0.48	0.358(0.221-0.563)	<0.001****	
**Age**									0.381
≤65	257	1.76	0.65	19	1.36	0.38	0.274(0.096-0.685)	0.010**	
(65,70]	113	1.64	0.59	27	1.30	0.55	0.328(0.136-0.721)	0.008**	
(70,75]	88	1.66	0.62	17	1.35	0.35	0.335(0.101-0.920)	0.050	
>75	65	1.60	0.62	16	1.54	0.56	0.837(0.308-2.046)	0.710	
**Gender**									0.439
Male	258	1.67	0.60	48	1.40	0.49	0.419(0.223-0.751)	0.005**	
Female	265	1.73	0.65	31	1.32	0.46	0.286(0.128-0.586)	0.001**	
**BMI(kg/m²)**									0.693
<18.5	50	1.47	0.48	6	1.42	0.46	0.791(0.095-4.329)	0.807	
[18.5,24.0)	282	1.70	0.57	50	1.40	0.51	0.349(0.186-0.628)	<0.001***	
[24.0,28.0)	139	1.84	0.76	19	1.36	0.43	0.291(0.106-0.683)	0.009**	
≥28	52	1.51	0.62	4	0.98	0.03	0.136(0.009-1.092)	0.096	
**Time from Onset to Admission (days)**									0.061
≤3	51	1.69	0.56	7	1.22	0.60	0.170(0.022-0.867)	0.053	
(3,7]	364	1.62	0.61	57	1.40	0.47	0.498(0.287-0.834)	0.010*	
(7,13]	77	1.97	0.69	12	1.26	0.47	0.060(0.008-0.287)	0.002**	
>13	31	1.93	0.64	3	1.58	0.33	0.340(0.023-2.645)	0.354	
**Hypertension History**									0.772
N	403	1.71	0.59	54	1.40	0.47	0.351(0.192-0.615)	<0.001***	
Y	120	1.66	0.74	25	1.30	0.50	0.406(0.176-0.841)	0.023*	
**Diabetes History**									0.803
N	462	1.72	0.62	67	1.40	0.47	0.353(0.208-0.577)	<0.001****	
Y	61	1.53	0.65	12	1.24	0.54	0.419(0.104-1.260)	0.166	
**Cardiovascular Disease History**									0.732
N	487	1.69	0.62	73	1.37	0.48	0.365(0.222-0.584)	<0.001****	
Y	36	1.77	0.73	6	1.35	0.47	0.260(0.026-1.306)	0.173	
**Cerebrovascular Disease History**									0.744
N	477	1.71	0.63	71	1.40	0.47	0.368(0.220-0.594)	<0.001****	
Y	46	1.58	0.63	8	1.16	0.51	0.285(0.054-1.093)	0.094	
**Chronic Hepatitis B History**									0.889
N	502	1.71	0.63	76	1.38	0.48	0.354(0.216-0.561)	<0.001****	
Y	21	1.47	0.53	3	1.28	0.32	0.432(0.018-5.202)	0.544	
**Suspicious Hypothyroidism**									0.111
N	494	1.70	0.63	78	1.36	0.47	0.345(0.211-0.547)	<0.001****	
Y	29	1.73	0.59	1	2.34	NA	5.448(0.178-Inf)	0.334	
**Smoking History**									0.446
N	435	1.70	0.64	64	1.35	0.47	0.328(0.190-0.543)	<0.001****	
Y	88	1.68	0.60	15	1.49	0.52	0.526(0.167-1.422)	0.241	
**Drinking History**									0.840
N	424	1.71	0.64	64	1.37	0.46	0.350(0.204-0.577)	<0.001****	
Y	99	1.67	0.59	15	1.39	0.55	0.396(0.127-1.103)	0.092	
**Statin History**									0.529
N	483	1.71	0.61	68	1.38	0.49	0.347(0.205-0.567)	<0.001****	
Y	40	1.61	0.80	11	1.32	0.44	0.524(0.143-1.428)	0.265	

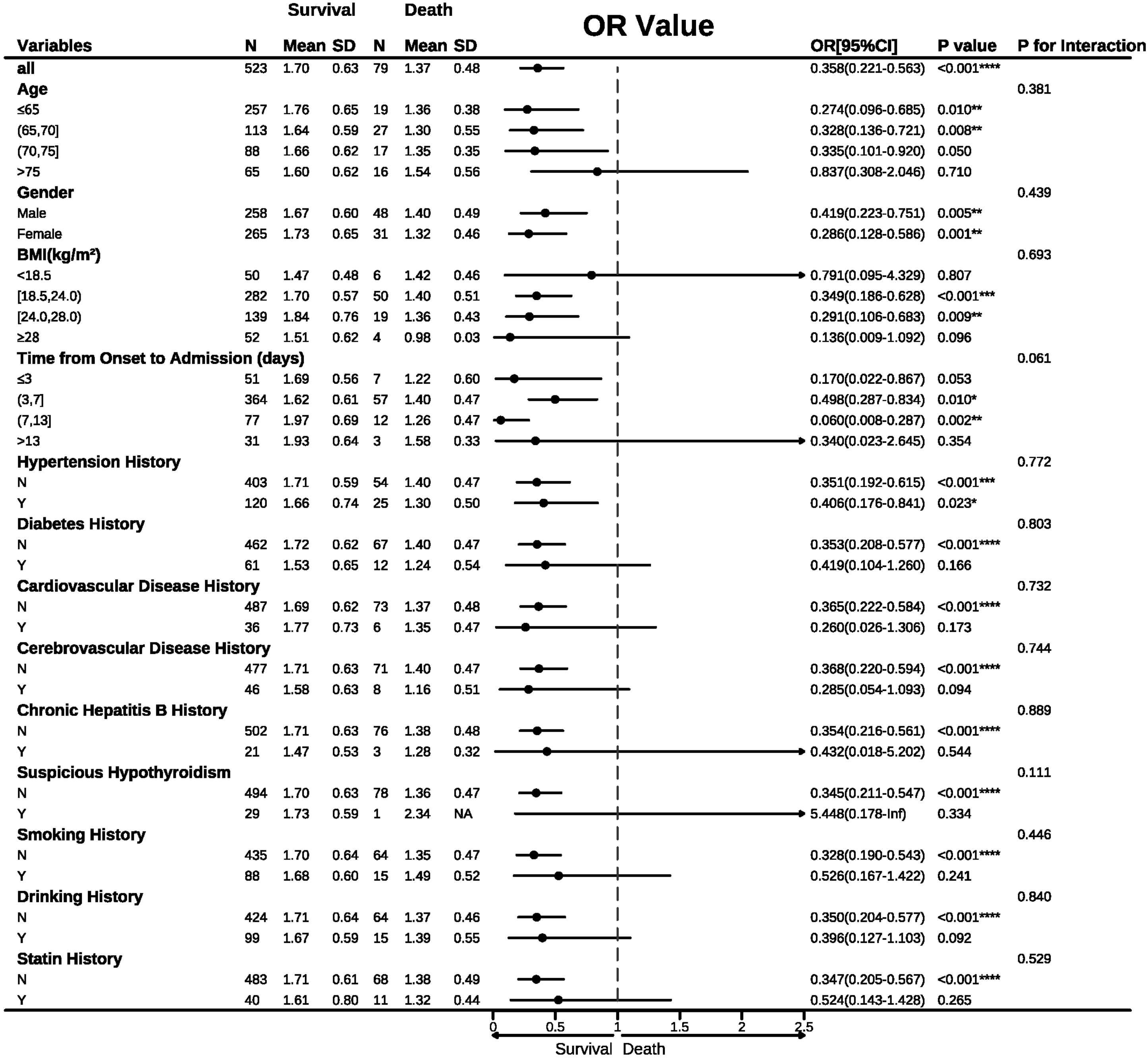

Figure S3. Forest Plot. of Subgroup Analysis. The subgroup analysis showed the interference of confounders on the correlation between LDL-C and mortality in SFTS. Logistic regression tests are first conducted within each subgroup. Subsequently, a likelihood ratio test is used to determine if the interactions are significant.

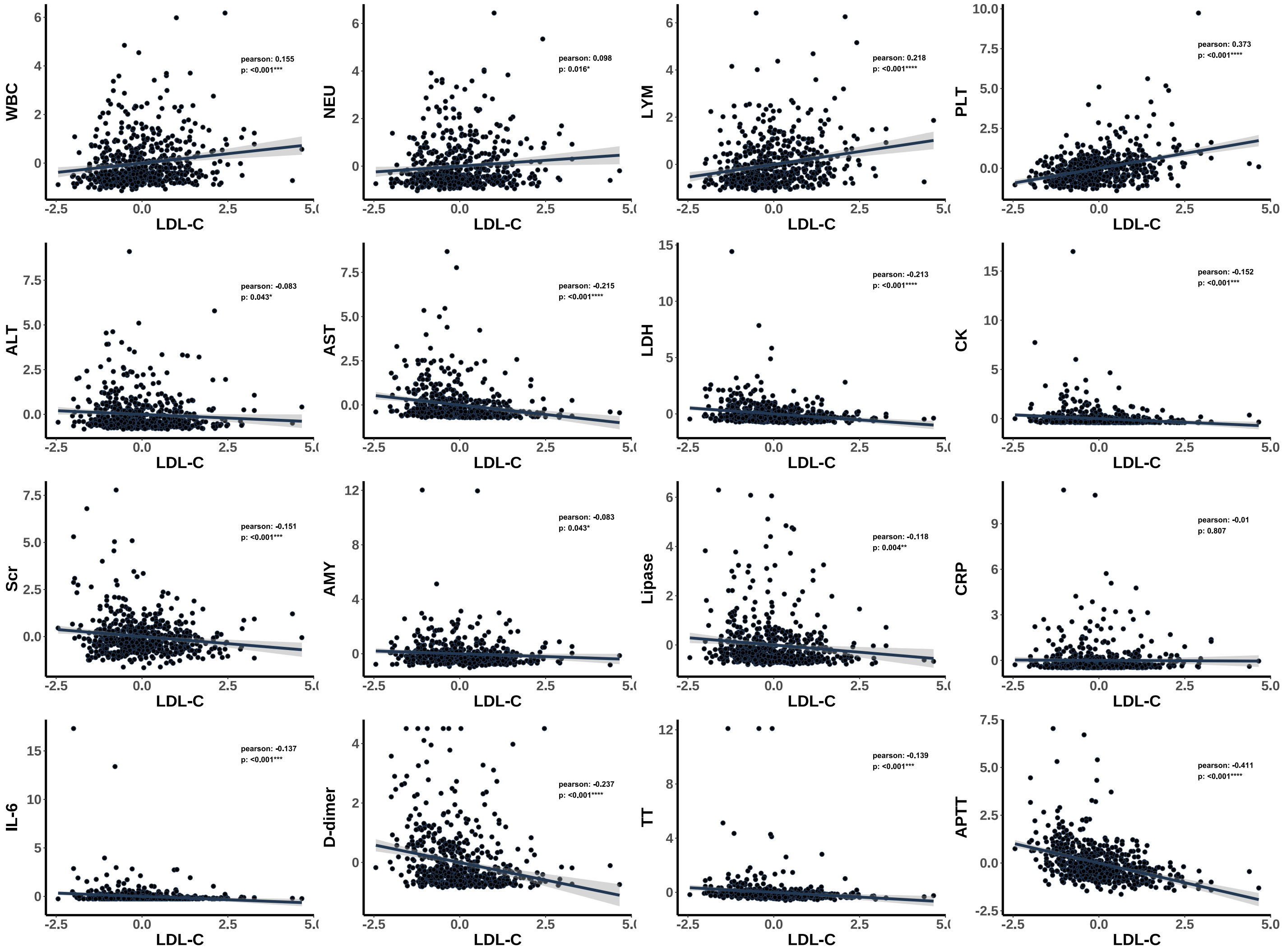

Figure S4. Pearson Correlation Plot. Before conducting mediation effect analysis, it is necessary to first perform correlation tests between LDL-C and potential mediators. Only variables that exhibit significant correlation are considered for further mediation effect analysis. Prior to conducting the correlation tests, all data are standardized to eliminate the impact of differing scales.

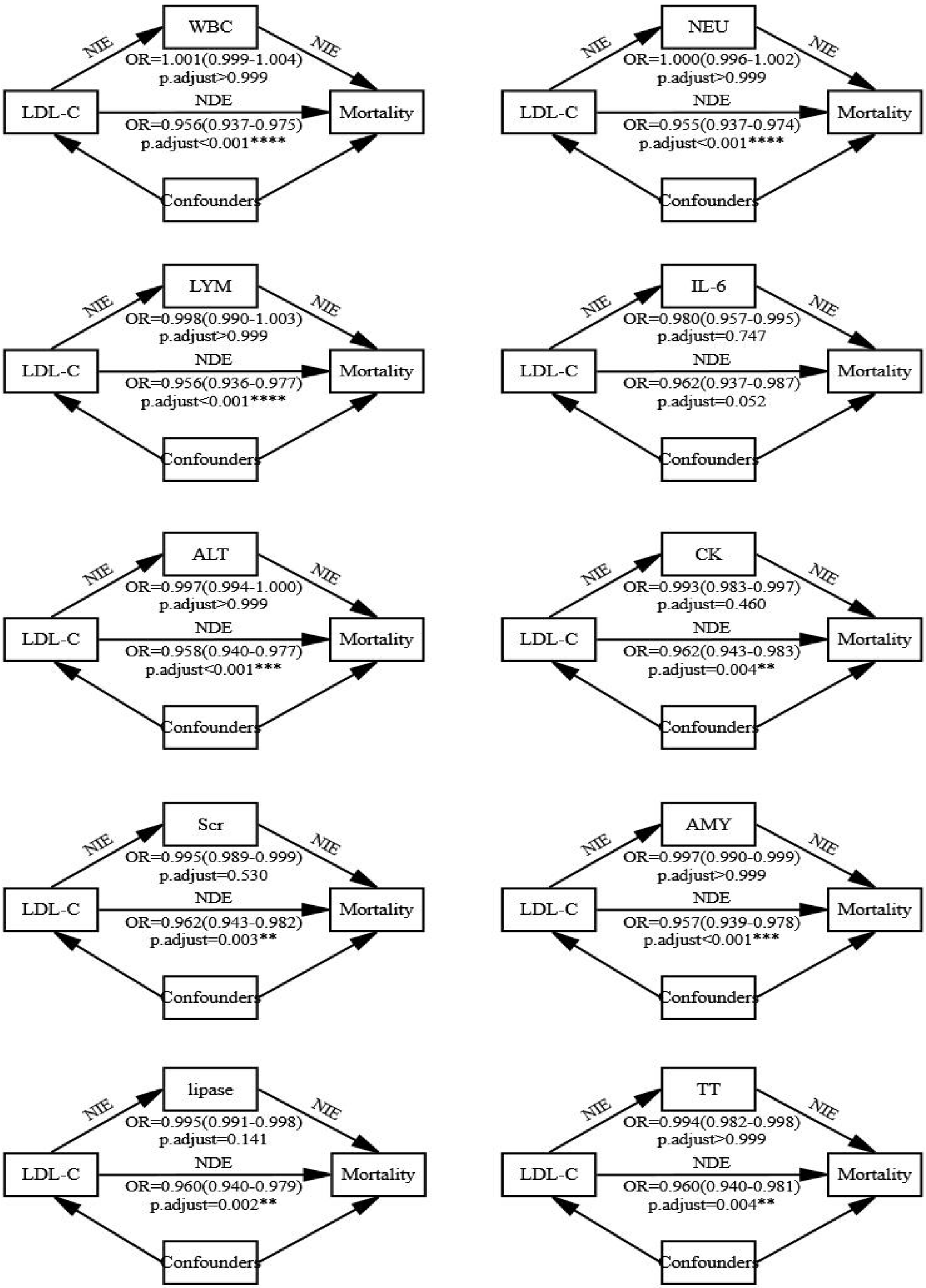

Figure S5. Mediation Effect Diagram. Ten processes with no significant mediation effects are displayed in the figure, which include WBC, NEU, LYM, IL-6, ALT, CK, Scr, AMY, lipase, and TT. The p.adjust for the NIE of all these processes is greater than 0.05. NIE, natural indirect effect; NDE, natural direct effect. For detailed values, please refer to Table S3.

Table S1. Prior Statin Medication Usage Among SFTS Patients.

Medical History	Statin Medication	Outcome: Survival (n=523)	Outcome: Death (n=79)	P value
**Hypertension**				
N(n=457)	N(n=447)	395(75.53%)	52(65.82%)	0.326
	Y(n=10)	8(1.53%)	2(2.53%)	
Y(n=145)	N(n=104)	88(16.83%)	16(20.25%)	
	Y(n=41)	32(6.12%)	9(11.39%)	
**Diabetes**				
N(n=529)	N(n=501)	441(84.32%)	60(75.95%)	0.161
	Y(n=28)	21(4.02%)	7(8.86%)	
Y(n=73)	N(n=50)	42(8.03%)	8(10.13%)	
	Y(n=23)	19(3.63%)	4(5.06%)	
**Cardiovascular Diseases**				
N(n=560)	N(n=532)	465(88.91%)	67(84.81%)	0.087
	Y(n=28)	22(4.21%)	6(7.59%)	
Y(n=42)	N(n=19)	18(3.44%)	1(1.27%)	
	Y(n=23)	18(3.44%)	5(6.33%)	
**Cerebrovascular Diseases**				
N(n=548)	N(n=516)	452(86.42%)	64(81.01%)	0.113
	Y(n=32)	25(4.78%)	7(8.86%)	
Y(n=54)	N(n=35)	31(5.93%)	4(5.06%)	
	Y(n=19)	15(2.87%)	4(5.06%)	

Notes: The *p* values were calculated by Mantel-Haenszel chi-squared test.

Table S2. LDL-C Time Trends within 28 Days after Admission.

Hospitalized Days	(0,2]	(2,4]	(4,7]	(7,11]	(11,14]	(14,21]	(21,28]
**Survival Group vs Death Group**							
Survival Group	1.61(1.26-2.10), N=502	1.74(1.28-2.21), N=209	2.10(1.56-2.61), N=379	2.30(1.84-2.79), N=343	2.14(1.73-2.74), N=141	2.11±0.76, N=90	1.95±0.85, N=32
Death Group	1.33(1.00-1.70), N=78	1.21(0.86-1.52), N=46	1.12(0.84-1.43), N=38	1.24(0.71-1.84), N=19	1.27(0.92-2.17), N=11	1.77±1.33, N=6	0.71±0.38, N=3
P value	<0.001****	<0.001****	<0.001****	<0.001****	0.022*	0.567	0.008**
**Non-critical Group vs Critical Group**							
Non-critical Group	1.62(1.29-2.11), N=474	1.78(1.29-2.22), N=193	2.15±0.75, N=360	2.40±0.72, N=315	2.38±0.71, N=117	2.18(1.82-2.70), N=64	2.22±0.76, N=11
Critical Group	1.20(0.94-1.68), N=28	1.36(1.04-1.68), N=16	1.45±0.58, N=19	1.82±0.62, N=28	1.53±0.63, N=24	1.50(1.08-2.07), N=26	1.82±0.87, N=21
P value	0.002**	0.030*	<0.001****	<0.001****	<0.001****	<0.001***	0.191

Notes: The *p* values were calculated by wilcoxon rank-sum test.

Table S3. Mediation Analysis of LDL-C on SFTS Mortality.

Variable	Effect Type	OR	p.raw	p.adjust
**Inflammatory Indicators**				
WBC	Indirect (ab)	0.999(0.993-1.002)	0.634	>0.999
	Direct (c')	0.956(0.937-0.975)	<0.001****	<0.001***
	Total (c)	0.955(0.937-0.973)	<0.001****	<0.001****
NEU	Indirect (ab)	1.000(0.996-1.002)	0.756	>0.999
	Direct (c')	0.955(0.937-0.974)	<0.001****	<0.001****
	Total (c)	0.955(0.937-0.973)	<0.001****	<0.001****
LYM	Indirect (ab)	0.998(0.990-1.003)	0.655	>0.999
	Direct (c')	0.956(0.936-0.977)	<0.001****	<0.001***
	Total (c)	0.954(0.937-0.973)	<0.001****	<0.001****
IL-6	Indirect (ab)	0.980(0.957-0.995)	0.050*	0.747
	Direct (c')	0.962(0.937-0.987)	0.003**	0.052
	Total (c)	0.943(0.910-0.969)	<0.001***	0.004**
**Hepatic Function Indicators**				
ALT	Indirect (ab)	0.997(0.994-1.000)	0.110	>0.999
	Direct (c')	0.958(0.940-0.977)	<0.001****	<0.001***
	Total (c)	0.955(0.936-0.973)	<0.001****	<0.001****
AST	Indirect (ab)	0.989(0.982-0.993)	<0.001***	0.010**
	Direct (c')	0.966(0.948-0.989)	0.001**	0.020*
	Total (c)	0.956(0.937-0.975)	<0.001****	<0.001***
**Cardiac Function Indicators**				
CK	Indirect (ab)	0.993(0.983-0.997)	0.031*	0.460
	Direct (c')	0.962(0.943-0.983)	<0.001***	0.004**
	Total (c)	0.954(0.933-0.974)	<0.001****	<0.001***
LDH	Indirect (ab)	0.979(0.962-0.989)	0.002**	0.026*
	Direct (c')	0.977(0.957-1.002)	0.052	0.786
	Total (c)	0.957(0.932-0.978)	<0.001***	0.005**
**Renal Function Indicators**				
Scr	Indirect (ab)	0.995(0.989-0.999)	0.035*	0.530
	Direct (c')	0.962(0.943-0.982)	<0.001***	0.003**
	Total (c)	0.957(0.937-0.974)	<0.001****	<0.001***
**Pancreatic Function Indicators**				
AMY	Indirect (ab)	0.997(0.990-0.999)	0.229	>0.999
	Direct (c')	0.957(0.939-0.978)	<0.001****	<0.001***
	Total (c)	0.955(0.935-0.974)	<0.001****	<0.001***

Lipase	Indirect (ab)	0.995(0.991-0.998)	0.009**	0.141
	Direct (c')	0.960(0.940-0.979)	<0.001***	0.002**
	Total (c)	0.955(0.936-0.974)	<0.001****	<0.001***
**Coagulation Function Indicators**				
PLT	Indirect (ab)	0.978(0.963-0.990)	0.002**	0.027*
	Direct (c')	0.969(0.950-0.991)	0.005**	0.079
	Total (c)	0.948(0.930-0.967)	<0.001****	<0.001****
TT	Indirect (ab)	0.994(0.982-0.998)	0.162	>0.999
	Direct (c')	0.960(0.940-0.981)	<0.001***	0.004**
	Total (c)	0.955(0.933-0.974)	<0.001****	<0.001***
APTT	Indirect (ab)	0.963(0.945-0.977)	<0.001****	<0.001****
	Direct (c')	0.997(0.974-1.025)	0.816	>0.999
	Total (c)	0.960(0.937-0.981)	<0.001***	0.004**
D-dimer	Indirect (ab)	0.987(0.979-0.993)	<0.001***	0.011*
	Direct (c')	0.971(0.952-0.990)	0.004**	0.066
	Total (c)	0.959(0.939-0.977)	<0.001****	<0.001***

Notes: The *p* values were calculated using bootstrap with 1000 iterations.

Table S4. Laboratory test abbreviations, Units and Normal Range.

Abbreviations	Full Name	Units	Normal Range
**Inflammatory Indicators**			
WBC	Count of white blood cells	10^9/L	3.50-9.50
NEU	Count of neutrophils	10^9/L	2.00-7.00
LYM	Count of lymphocytes	10^9/L	0.80-4.00
CPR	C-reactive protein	mg/L	0-8
IL-6	Interleukin-6	pg/mL	≤5.40
**Hepatic Function Indicators**			
ALT	Alanine transaminase	U/L	9-50
AST	Aspartate transaminase	U/L	15-45
**Cardiac Function Indicators**			
CK	Creatine kinase	U/L	50-310
LDH	Lactate dehydrogenase	U/L	120-250
**Renal Function Indicators**			
Scr	Serum creatinine	μmol/L	57.0-111.0
**Pancreatic Function Indicators**			
AMY	Serum amylase	U/L	35-135
Lipase	Serum lipase	U/L	13.0-60.0
**Coagulation Function Indicators**			
PLT	Count of platelet	10^9/L	125-350
TT	Thrombin time	s	14.0-21.0
APTT	Activated partial thromboplastin time	s	28.0-45.0
D-dimer	D-dimer	mg/L	≤0.50
**Serum Lipid Profiles**			
TG	Triglycerides	mmol/L	<1.70
Total Cholesterol	Total Cholesterol	mmol/L	≤5.18
HDL-C	High density lipoprotein cholesterol	mmol/L	>1.04
LDL-C	Low density lipoprotein cholesterol	mmol/L	<3.37

Notes: The normal ranges are derived from the clinical laboratory test forms of the Shandong Public Health Clinical Center.
